# Hepatoid adenocarcinoma of the stomach with metastatic choriocarcinoma of the liver: A case report of a rare subtype of gastric cancer with a complex treatment course

**DOI:** 10.3389/fsurg.2022.968891

**Published:** 2022-09-09

**Authors:** Qiyang Zhou, Yudi Zhou, Yiming Ouyang, Weichang Chen, Xiaojun Zhou

**Affiliations:** ^1^ Department of General Surgery, The First Affiliated Hospital of Soochow University, Suzhou, China; ^2^ Department of Gastroenterology, The First Affiliated Hospital of Soochow University, Suzhou, China

**Keywords:** gastric hepatoid adenocarcinoma, hepatic choriocarcinoma, chemotherapy, neoadjuvant therapy, radical surgery, case report

## Abstract

Gastric hepatoid adenocarcinoma and hepatic choriocarcinoma are rare diseases in clinical settings, and the case we report here is a combination of both. A 66-year-old woman presented with a chief complaint of abdominal discomfort. The patient was examined using gastroscopy and computed tomography (CT) scan, and these revealed an irregular surface ulcer on the wall of the gastric antrum. A mass, 2.0 cm in diameter, was found in the liver in April 2020. The endoscopic biopsy findings were consistent with a diagnosis of moderately to poorly differentiated hepatoid adenocarcinoma. She was then referred to our hospital for further treatment. Initially, neoadjuvant therapy was initiated for the patient. The CT scan showed that the liver metastases had progressed; hence, surgery was performed. Postoperative pathology showed that the gastric lesions were mostly hepatoid adenocarcinoma with no choriocarcinoma, while the liver lesions comprised approximately 10% hepatoid adenocarcinoma and 90% choriocarcinoma. One month later, the patient developed tumor recurrence in the liver as observed on CT imaging. Subsequently, a variety of chemotherapy regimens were tried with no obvious results. The patient eventually developed multiple organ metastasis and died in July 2021. The overall survival was 16 months. Based on findings from this case report, it appears that initial neoadjuvant therapy was not effective and radical surgery may be the best treatment for patients with hepatoid adenocarcinoma of the stomach.

## Introduction

Hepatoid adenocarcinoma of the stomach (HAS) is a rare type of primary gastric malignant neoplasm that exhibits both adenocarcinomatous and hepatocellular differentiation and mostly produces alpha-fetoprotein (AFP). It accounts for approximately 1% of all gastric cancers. The stomach is the most commonly affected organ, and it often occurs in the antrum accompanied by vascular invasion, lymph node metastasis, and liver metastasis (1–3). Choriocarcinoma, on the other hand, is prone to rapid hematogenous metastases, and the first clinical manifestations are often metastatic lesions (4). The characteristic laboratory finding in patients with choriocarcinoma is an elevated serum human chorionic gonadotropin (hCG) level (5). Due to the rarity and ease of metastasis associated with these neoplasms, timely diagnosis and proper treatment are of significant importance. Herein, we report a case of HAS with liver metastasis, in which the pathologic diagnosis was choriocarcinoma (90%) combined with hepatoid adenocarcinoma (10%). Furthermore, we discuss this case in the context of other similar studies, which ultimately highlight the intricacy of HAS and the need for sound treatment guidelines.

## Case presentation

**Figure 1:**
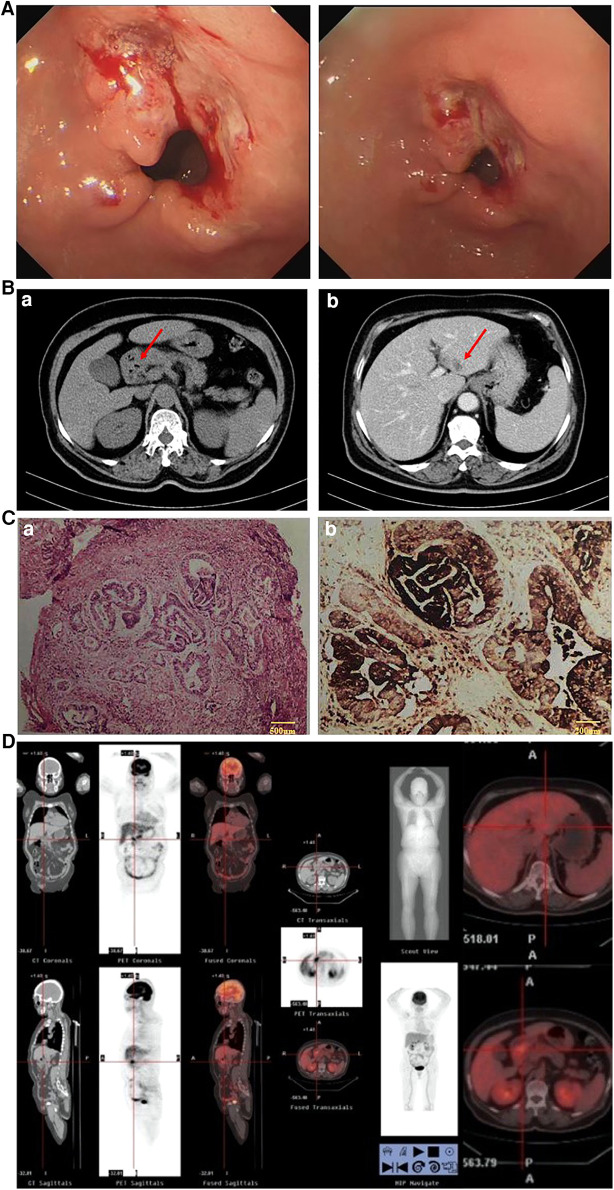
(**A**) Gastroscopy (April 1, 2020): gastric antrum ulcer. (**B**) CT (April 1, 2020): (a) Gastric antrum tumor; (b) Abnormal low density of left lateral lobe of the liver, considering metastasis. (**C**) Results of endoscopic biopsy: (a) HE staining; (b) AFP staining. (Gastric antrum) Moderately to poorly differentiated hepatoid adenocarcinoma. Immunopathology: AFP(+), HER-2(+), Ki-67(40%), PD-L1(22C3)(−), PD-1(−), CD4(+), CD8(+), MLH1(+), PMS2(+), MSH6(+), MSH2(+). (**D**) PET-CT examination: The masses of gastric antrum and left liver were hypermetabolic.

This was a case of a 66-year-old female patient with a complaint of abdominal discomfort for a month with no other associated symptoms. Physical examination revealed no pathological findings. Gastroscopy and computed tomography (CT) examinations revealed an irregular surface ulcer on the wall of the gastric antrum (Figure 1A) and a mass of 2.0 cm in diameter was found in the liver (Figure 1B). The pathological diagnosis from endoscopic biopsy was consistent with a moderately to poorly differentiated hepatoid adenocarcinoma (Figure 1C). PET-CT examination was then performed, and this showed that the stomach and liver masses were hypermetabolic (Figure 1D).

Laboratory tests showed that the blood cell and biochemical values were almost within the normal range. However, serum AFP was dramatically elevated (>2,000 ng/ml). Further examination revealed the remaining tumor markers, including the carbohydrate antigen 199 (CA199) >1200 U/ml, carcinoembryonic antigen 38.17 ng/ml, and hCG 795.14 mIU/ml.

Based on the guidelines of the Chinese Society of Clinical Oncology (CSCO), the disease was classified as advanced gastric cancer with liver metastasis, and the patient underwent SOX chemotherapy regimen, which is the first-line treatment recommendation, consisting of oxaliplatin, gimeracil, and oteracil potassium capsules.

**Figure 2:**
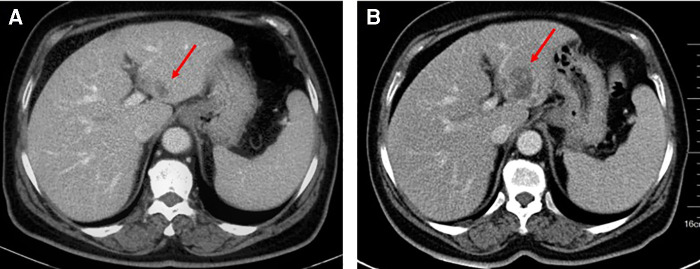
Ct examination: (**A**) April 1, 2020. (**B**) June 17, 2020. The mass of the left liver was more advanced than before.

**Figure 3:**
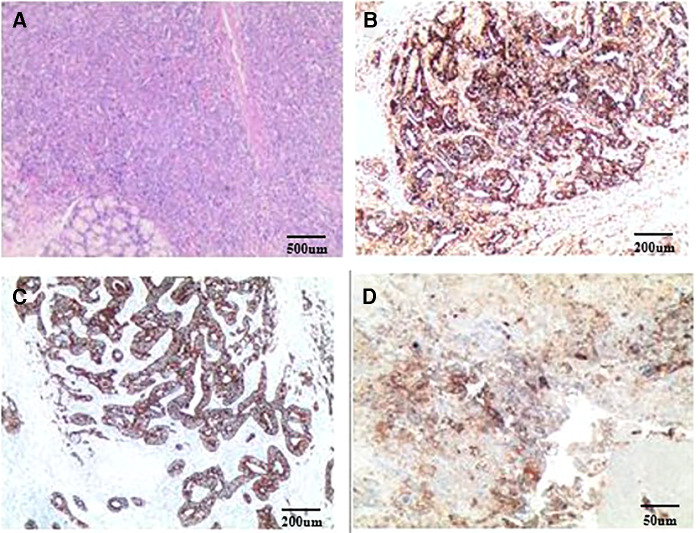
Postoperative pathology: (**A**) HE staining; (**B**) AFP staining; (**C**) CK staining; (**D**) PD-L1 staining. Routine pathology: (Stomach) Moderately to poorly differentiated adenocarcinoma, focal features of hepatoid adenocarcinoma, small necrosis, tumor invasion, tumor thrombus in the vessel; (Right liver nodule S5) cancer metastasis; (Left lateral liver) cancer metastasis, with massive necrosis, bleeding, no cancer accumulation in the liver resection margin, while the liver lesions were about 10% of hepatoid adenocarcinoma and 90% were choriocarcinoma. Immunopathology: (Stomach): CK(+), AFP(+), Glypican-3(+), MSH2(+), MSH6(+), MLH1(+), PMS2(+), MSS, Her-2(1+), Ki-67(+, 95%), PD-L1(22C3) (tumor cell: 1%–5%; microenvironment immune cells: +, 20%–30%), CD117(+), Hepa-1(−), CK7(−), CK5/6(−), P40(−), PD-1(−), CD5(−), Vimentin(−), EVB(−); (Liver): CK7(+), Her-2(2+), Ki67(+, 95%), PD-L1(22C3)(+, >50%), MLH1(+), MSH2(+), MSH6(+), PMS2(+), MSS, AFP(−), Hepa-1(−), CD5(−), and EBV(−).

After three cycles of chemotherapy, CT revealed progression of liver metastasis (Figure 2). The patient had leucopenia during chemotherapy and chose surgery for the subsequent treatment. Radical gastrectomy, hepatic left lateral lobectomy, and resection of the metastatic nodule in hepatic segment V were performed. Postoperative pathology (Figure 3) showed that the stomach mass was hepatoid adenocarcinoma, while the mass in the liver was approximately 10% hepatoid adenocarcinoma and 90% choriocarcinoma, with lymphatic metastasis (7/26).

**Figure 4:**
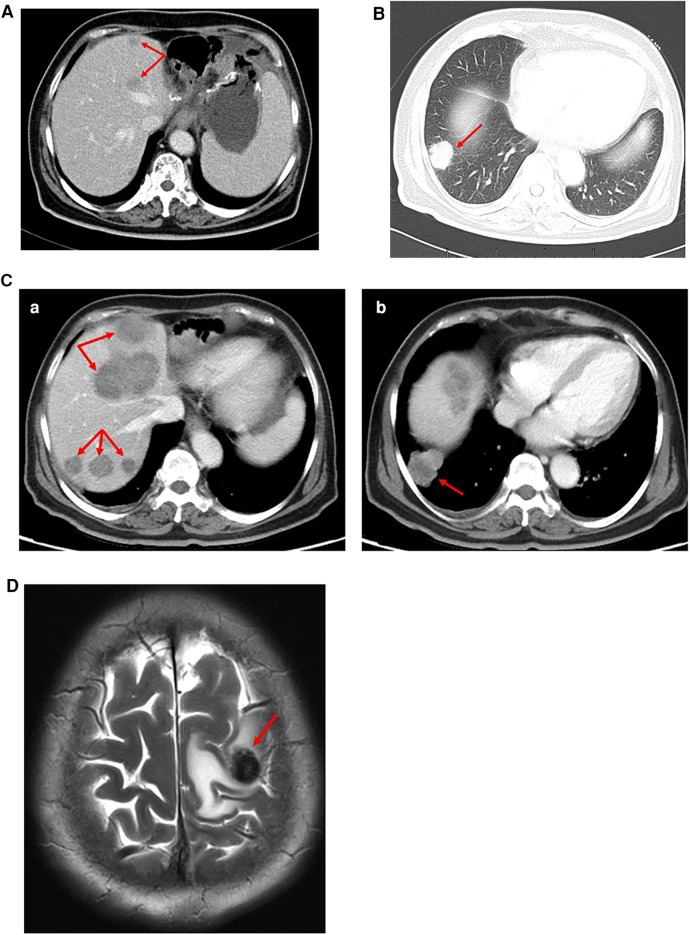
(**A**) CT examination (July 28, 2020): many slightly low-density lesions in the liver, considering metastasis. (**B**) CT examination (September 14, 2020): Right pulmonary nodule, considering metastasis. (**C**) CT examination (November 19, 2020): (a) Multiple low-density liver metastases (more and larger than before). (b) The metastatic nodules in the lower lobe of the right lung were larger than before. (**D**) MR examination (March 18, 2021): Brain metastasis.

One month later, CT imaging revealed liver metastasis and recurrence (Figure 4A). Considering the poor effect of the original chemotherapy regimen, we chose the DS chemotherapy regimen, which consisted of paclitaxel, gimeracil, and oteracil potassium capsules.

After two cycles of the chemotherapy regimen, on September 14, 2020, CT reexamination showed that liver and lung metastases occurred (Figure 4B). We changed the chemotherapy regimen again. Sintilimab, paclitaxel, and regorafenib were selected for treatment. This chemotherapy regimen was administered three times.

Two months later, CT examination showed that the liver and lung metastases were more and larger than before (Figure 4C). We chose to replace the former chemotherapy scheme with FOLFOX chemotherapy regimen (oxaliplatin, folinic acid calcium salt hydrate, and 5-FU) and sintilimab as a new treatment.

On March 18, 2021, the magnetic resonance (MR) scan showed that the patient had developed brain metastases (Figure 4D). Then, she was treated with etoposide and KN046 (PD-L1/CTLA-4 bispecific antibody) and intracranial radiotherapy.

Overall, a variety of chemotherapy regimens were tried, and the patient developed systemic multiple organ metastasis. During the whole chemotherapy period, the indexes of tumor markers remained at high levels, including AFP, CA199, and hCG.

Finally, the patient suffered from repeated low fever, trilineage cytopenia, and her physical condition gradually worsened. She eventually rejected treatment and died in July 2021.

## Discussion

With an increasing number of articles reporting cases of HAS, the diagnosis of hepatoid adenocarcinoma is no longer a problem through laboratory examination, gastroscopy, and pathological biopsy. HAS is a rare type of primary gastric malignant neoplasm and it is accompanied by vascular invasion, lymph node metastasis, and liver metastasis. Extragenital choriocarcinomas, on the other hand, are less common, often exist with other carcinomas, and tend to occur in midline organs (4). Fernández Alonso et al. reported primary hepatic choriocarcinoma for the first time in 1992 (6). Subsequently, most patients with hepatic choriocarcinoma were reported from China (7, 8). Conversely, the histopathogenesis of extragonadal choriocarcinomas remains controversial. Some authors have reported that normal cells develop directly into choriocarcinoma (9), whereas others have reported that retrodifferentiation from pre-existing adenocarcinoma results in choriocarcinoma (10). In this case, the patient was examined through gastroscopy and biopsy and these confirmed the diagnosis of hepatoid adenocarcinoma. Since CT showed evidence of a lesion in the liver, liver metastasis was suspected. Laboratory tests showed that in addition to the increase in AFP level (beyond 2,000 ng/ml), hCG was also significantly elevated to 795.14 mIU/ml. However, the reason for the increase in the hCG index was unclear at this point. To ensure that metastases had not occurred at other primary sites, PET-CT examination was performed and this revealed no obvious tumor in other organs. PET-CT also showed that the tumors in the stomach and liver were hypermetabolic. Therefore, we diagnosed the patient as having hepatoid gastric adenocarcinoma with liver metastasis. Postoperative pathological examinations showed that the gastric lesions were comprised mostly of hepatoid adenocarcinoma and no choriocarcinoma, while the liver lesions were approximately 10% hepatoid adenocarcinoma and 90% choriocarcinoma. In fact, we were not sure whether the liver choriocarcinoma was the primary or differentiated tumor after metastasis. Liu et al. pointed out that metastatic lesions will differentiate when they metastasize to other organs (10), and the experience in this case seemed to agree with this.

The patient's condition was diagnosed as HAS with liver metastasis; hence neoadjuvant therapy was initiated first. To date, there is still no clear criterion for the treatment of HAS. Some studies have reported radical surgery as the main treatment option for patients with HAS (11, 12). However, the specific regimen of neoadjuvant or adjuvant therapy for HAS remains unclear. A recent systematic review concluded that cisplatin-based chemotherapy is the most efficient first-line systemic treatment in advanced situations, with a clinical response observed in 75% of the patients (13). Arakawa et al. recently reported a significant clinical response to ramucirumab monotherapy in a metastatic HAS (mHAS) patient with chemotherapy-resistant recurrent disease (14). The introduction of molecular targeted therapy has also brought hope to HAS patients. Hepatic choriocarcinoma, on the other hand, has no clear treatment strategy; although surgery and chemotherapy have been reported, none of the patients treated with these had good prognosis (8, 15, 16). Neoadjuvant therapy was the initial treatment option employed in this case. We used the SOX chemotherapy regimen, which consisted of oxaliplatin, gimeracil, and oteracil potassium capsules, a treatment modality not reported in the earlier literature referred; however, this is the first-line treatment for advanced metastatic gastric cancer according to our guidelines. The patient underwent simultaneous whole-genome sequencing, but the results showed that there was no new targeted drug recommended for the patient. The neoadjuvant chemotherapy did not achieve obvious results. We are puzzled whether the next treatment plan is to continue the second-line treatment plan or surgery. At that time, we assessed that the liver and stomach lesions could still be resected by operation through CT imaging. It is very difficult for us to continue to choose the second-line chemotherapy or surgery. However, the patient and her family had a strong desire for surgery; hence, surgery was performed. Nonetheless, the patient's liver tumor recurred one month after the surgery and a variety of chemotherapy regimens were tried after that, including targeted medicine and bispecific antibody with no obvious results. The disease was not controlled, multiple organ metastasis occurred, and, eventually, the patient died.

There is no standard treatment for mHAS at present. This case report discusses its clinical treatment and prognosis and helps get further knowledge and understanding of mHAS and the treatment of this rare aggressive tumor. However, a limitation of this case report may be that molecular studies were not performed; thus, HER2 gene amplification and overexpression and EGFR, KRAS, and BRAF mutation-associated tumorigenesis and gastric carcinomas that may respond to specific treatments were not able to be explored in this case report.

## Conclusion

In summary, HAS and hepatic choriocarcinoma are rare and aggressive diseases; the combination of the two is even rarer, and the diagnosis and treatment are full of challenges. Although radical surgery may be the best treatment for HAS patients, the specific systemic chemotherapy principles for patients with advanced metastasis resulting from HAS are still flawed. There is an urgent need for a large number of clinical reports to improve the understanding of HAS and hepatic choriocarcinoma.

## Data Availability

The raw data supporting the conclusions of this article will be made available by the authors, without undue reservation.
